# Progress in the Application of Immune Checkpoint Inhibitor-Based Immunotherapy for Targeting Different Types of Colorectal Cancer

**DOI:** 10.3389/fonc.2021.764618

**Published:** 2021-11-23

**Authors:** Rui He, Yefang Lao, Wenyan Yu, Xiaohui Zhang, Min Jiang, Chunrong Zhu

**Affiliations:** ^1^ Department of Oncology, The First Affiliated Hospital of Soochow University, Suzhou, China; ^2^ Department of Oncology, Shanghai International Medical Center, Shanghai, China

**Keywords:** immunotherapy, colorectal cancer, immune checkpoint inhibitors, microsatellite, ICI combination therapy

## Abstract

Colorectal cancer (CRC), a common malignant disease, has the second highest mortality rate among all cancer types. Due to the diversity and heterogeneity of CRC, few effective treatment strategies have been developed in recent years, except for surgical resection. As immunotherapy has become a revolutionary treatment after surgery, along with chemoradiotherapy and targeted therapy, numerous basic research studies and clinical trials have been conducted on CRC. Therefore, immune checkpoint inhibitor (ICI) therapy has become the main anti-CRC immunotherapy method used at present. With the rapid development of biotechnology and cell research, an increasing number of monotherapy or combination therapy strategies using ICIs for CRC have been designed in recent years. Methods to classify and review ICI strategies for different types of CRC to better guide treatment are continuously investigated. However, the identification of why the ICIs would be more effective in targeting particular subtypes of CRC such as high microsatellite instability (MSI-H) is more important because of the different immune backgrounds in patients. This review intends to classify different subtypes of CRC and summarizes the basic and clinical studies on ICIs for each subtype of CRC currently available. In addition, we also attempt to briefly discuss the progress in immunotherapy methods other than ICI therapy, such as chemoimmunotherapy strategy, chimeric antigen receptor-modified T (CAR-T) cells, or immunotherapy based on oncolytic viruses. Finally, we provide a perspective on the development of immunotherapy in the treatment of CRC and attempt to propose a new systematic classification of CRC based on immunological strategies, which may improve guidance for the selection of immunotherapy strategies for different subtypes of CRC in the future.

## 1 Introduction

According to the latest global statistics, non-communicable diseases are still the leading cause of death worldwide, among which cancer is an important disease endangering human life. In 2018, the number of patients with a new cancer diagnosis reached more than 18 million, and 9.6 million patients died because of cancer, with continuous growth (1). As a life-threatening disease, colorectal cancer (CRC) was estimated to have the third highest incidence (6.1%) and the second highest mortality (9.4%) of all cancers. In America, in 2020, CRC remained a leading cause of cancer-related death, accounting for 40%–50% of all new diagnoses of breast, lung, and colorectal cancers (2, 3). Among the common sites of CRC, approximately 41% occur in the proximal colon, 22% in the distal colon, and 28% in the rectum (4, 5). Notably, 50% of patients will develop distant metastases known as metastatic CRC (mCRC), which has a high mortality rate. Therefore, new and effective treatment strategies for patients with CRC now need to be developed to reduce the mortality rate of CRC.

Immunotherapy has become the fourth largest cancer treatment program after surgery, chemoradiotherapy, and targeted therapy. The first clinical example of tumor immunotherapy dates to 1891, when Coley et al. accidentally observed a reduction in tumor volume by injecting *Streptococcus* into patients with inoperable osteosarcoma (6). Bacterial infection enhances the local immune response of tumor tissue, which induces activated immune cells such as T cells to destroy tumor cells, leading to the opening of a new chapter in cancer immunotherapy (6–8). In the past decade, immunotherapy using immune checkpoint inhibitors (ICIs) has attracted increasing attention due to its success in producing long-lasting responses to solid tumors such as melanoma and lung cancer (9). Since Le et al. found that patients with CRC and a deficient DNA mismatch repair (MMR) (dMMR) or high microsatellite instability (MSI) (MSI-H) benefit from ICI treatment in 2015 (10), ICIs such as programmed cell death 1/programmed cell death 1 ligand (PD-1/PD-L1) inhibitors and cytotoxic T lymphocyte (CTL) antigen 4 (CTLA4) inhibitors have been utilized to treat patients with CRC. However, although research suggests that different malignancies, such as melanoma, kidney cancer, bladder lung cancer, or Hodgkin’s disease, respond well to ICI immunotherapy, only a minor proportion of patients with mCRC benefit from it (10–13). With the rapid development of molecular biology and clinical trials concerning immunotherapy, more effective ICI strategies have been developed. In order to improve the effect of ICIs on CRC, more combination therapy strategies of ICIs and other drugs have become new research direction. In addition, immunotherapy methods other than ICIs, such as chimeric antigen receptor-modified T (CAR-T) cells or immunotherapy based on oncolytic viruses, have emerged rapidly in recent years. Cellular immunotherapy based on CAR-T cells has been successfully developed for the treatment of relapsed and refractory B-line lymphoblastic leukemia in recent years (14–16), which has encouraged the development of immunotherapy for CRC (17). Immunotherapy, a new and powerful antitumor therapy, would become an alternative treatment strategy for CRC patients.

**Figure 1:**
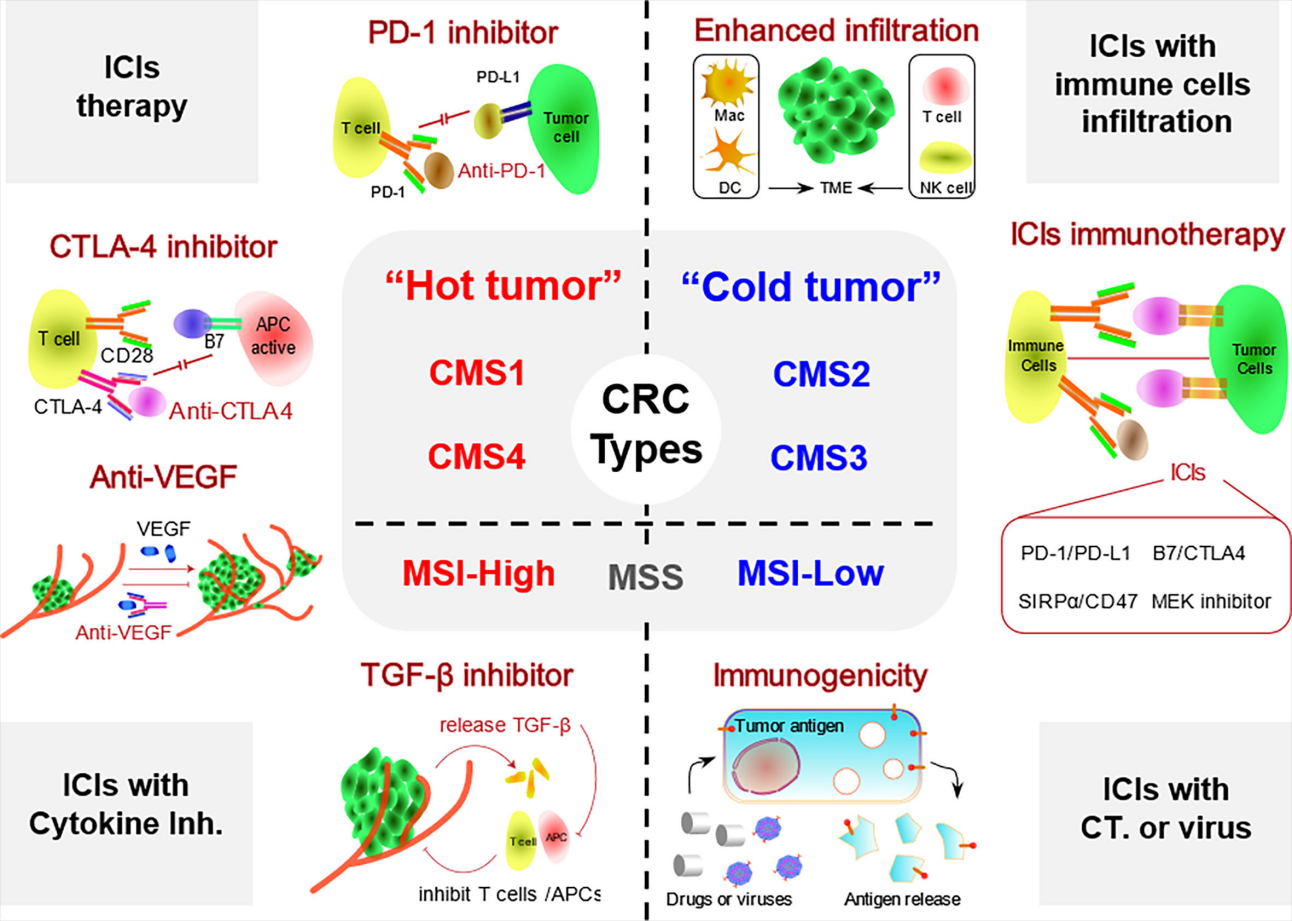
Different types of ICI-based immunotherapy strategies for patients with CRC. CRC is divided into hot and cold tumor subtypes. Hot CRC mainly includes the dMMR/MSI-H, CMS1, and CMS4 subtypes, while cold CRC includes the pMMR/MSI-L, CMS2, and CMS3 subtypes. TME, tumor microenvironment; Inh., inhibitor; CT, chemotherapy; ICI, immune checkpoint inhibitor; CRC, colorectal cancer; dMMR, deficient DNA mismatch repair; MSI-H, high microsatellite instability; pMMR, proficient mismatch repair.

In most CRC patients, tumor metastasis is always present. However, only patients with advanced CRC presenting with dMMR and MSI-H subtypes respond to ICIs (18). In contrast, the effect of immunotherapy on subtypes that are proficient in MMR (pMMR) and have microsatellite stability (MSS) and low MSI (MSI-L) has not been clearly determined. In patients with CRC, a high tumor mutational burden (TMB) has become a marker of immunotherapeutic responsiveness, while a lack of immune cell infiltration has been identified as a cause of tumor immune resistance (9, 19–21). Recently, immunotherapy strategies have become more diversified for patients with CRC (22). For CRC, which has multiple molecular subtypes, a more important goal is to review the optimal effects of different immunotherapies on different subtypes of CRC, which might guide the selection of clinical treatment strategies for patients with CRC. Therefore, we summarized the methods for classifying CRC in this review, and then we discussed different types of currently available ICI therapy strategies for each subtype of CRC. In addition, we discussed the progress in other immunotherapies for CRC in addition to ICI immunotherapy. Finally, we also provided a perspective on the development of immunotherapy for the treatment of CRC, and we attempted to propose a new systematic classification of CRC based on immunological strategies, which may guide the selection of immunotherapy strategies for different subtypes of CRC in the future (**Figure 1**).

## 2 Immune Checkpoint Inhibitors for Suppressed T Cells and Classification Systems of Colorectal Cancer

The tumor microenvironment (TME), which is defined as tumor cells and the surrounding environment, includes tumor-related immune cells, blood vessels, cytokines, stroma, epidermal growth factor (EGF), transforming growth factor-beta (TGF-β), fibroblast growth factor (FGF), tumor necrosis factor-alpha (TNF-α), and other signaling molecules. The TME affects the occurrence and development of tumors (22–24). Among them, immune cells accurately monitor and remove tumor cells *in vivo* and play a crucial role in inhibiting tumor growth (25). However, the physical barrier, biochemical barrier, and physiological barrier produced by the TME induce a strong inhibitory effect on the immune system, resulting in the inability of immune cells to exert their antitumor activities and even promoting the occurrence of cancer (26). Therefore, the formation of an immunosuppressive TME is an important indicator of tumor deterioration (27, 28). Among the immune cells, T cells have a strong killing activity of tumor cells. However, abnormally high expression of immune checkpoint receptors such as PD-L1 in tumor cells inhibited the recognition and destruction of T cells. For reactivating T cells, the most mature method is to use ICIs to remove the immunosuppression effect of tumor cells and enable T cells to replay cytotoxicity. The strategy of immune regulation is expected to achieve the functional activation of the immune system in the TME without any external tools, only using the natural antitumor system of the organism, which is also the main advantage of immunotherapy (29).

As a type of cancer with high morbidity and mortality rates, more effective treatment strategies have not been developed for CRC, except for surgical resection or classical chemotherapy strategies such as FOLFOX. Therefore, the effective formulation of antitumor strategies for CRC is important to reduce mortality. The MMR/MSI system is the most important indicator of CRC classification and is used to develop treatment strategies. Microsatellites are tandem repeats of dozens of nucleotides, comprising one to six nucleotides as repeat units (30). MSI is a frameshift mutation of a microsatellite in tumor cells due to the insertion or deletion of repeated units (31, 32). The MMR system works to combat these errors by identifying and repairing DNA damage and correcting insertions, deletions, or mismatched bases that result from the error cycle that occurs during DNA replication (33–35). Regarding the MMR/MSI system, MMR is divided into dMMR and pMMR. Notably, dMMR is manifested as the absence of MMR proteins. When the MMR system is dysfunctional or mutated, these genetic errors are not corrected, thus allowing them to be permanently integrated into the tumor DNA, which is called MSI-H (31). However, MMR protein expression is normal in pMMR, which is mainly divided into MSI-L or MSS (36, 37). The dMMR/MSI-H subtype of CRC accounts for approximately 15% of all cases and 5% of mCRC cases (38–40). Due to the high mutation rate in dMMR/MSI-H, the tumor has high immunogenicity, enabling it to activate the antitumor effect of the immune system. Patients with dMMR/MSI-H are more responsive to ICI-based immunotherapy, which has sparked strong interest in immunotherapy for CRC. Therefore, the search for new and more effective immunotherapy strategies for the treatment of different CRC subtypes has become mainstream (**Table 1**).

**Table 1:** Ongoing clinical trials for immunotherapy in patients with CRC.

Ongoing clinical trials for immunotherapy in CRC patients					
Name	Strategy	Trial type	Experimental arm treatment dosage	Trial identifier	Possible targeted subtypes
**No. 1 checkpoint inhibitor**					
Nivolumab and ipilimumab	PD-1 and CTLA4 inhibitors	·Phase II trial	Nivolumab (480 mg every 4 weeks)	NCT04730544	Mainly effective for hot CRC (dMMR/MSI-H, CMS1, and CMS4), which has a high content of immune cells, especially T cells.
		·dMMR and/or MSI mCRC	+ ipilimumab (1 mg/kg every 6 weeks)		
Camrelizumab and apatinib	PD-L1 and VEGF inhibitors	·Phase II trial	Camrelizumab (200 mg intravenous (i.v.) every 3 weeks)	NCT04715633	
		·Locally advanced dMMR/MSI-H CRC	+ Apatinib (250 mg qd days 1–14)		
Toripalimab with or without celecoxib	PD-1 and COX inhibitors	·Phase I and II trials	·Toripalimab (3 mg/m^2^ on day 1, every 2 weeks)	NCT03926338	
		·Resectable non-metastatic	+ Celecoxib (oral 200 mg twice daily for 12 weeks)		
		·dMMR/MSI-H colorectal Cancer	·Toripalimab (3 mg/m^2^ on day 1, every 2 weeks)		
Cetuximab–avelumab	PD-1 and EGFR inhibitors	·Phase II trail ·Metastatic colorectal cancer	·Toripalimab (10 mg/kg every 2 weeks) + Cetuximab (400 mg/m^2^ or 250 mg/m^2^ at first dose)	NCT04561336	
**No. 2 enhancing tumor immunogenicity**					
LOAd703	Oncolytic adenovirus	·Phase I and II trials	LOAd703 oncolytic adenovirus administered	NCT03225989	Oncolytic virus is effective against all CRC types, as well as low immunogenicity subtypes like pMMR/MSS.
		·Digestive tract cancers (colorectal cancer)	+ standard of care chemotherapy or gemcitabine		
TBio-6517 and pembrolizumab	Oncolytic virus and PD-1 inhibitor	·Phase I and II trials	TBio-6517, given intratumorally, alone or in combination with pembrolizumab (beginning at day 8 *via* i.v. infusion every 3 weeks)	NCT04301011	
		·Solid tumors (colorectal cancer)			
mDC3 vaccine	Active DCs and T cells	·Phase I trial	Receive the vaccine and be followed per the schedule of procedures.	NCT03730948	Mainly for hot CRC, which could directly increase TAAs.
		·Colorectal carcinoma			
NA DC vaccine and nivolumab	Active DCs and PD-1 inhibitor	·Phase II and III trials	NA DC vaccine every 2 weeks at a dose of 3–5 million cells	NCT04912765	
			+ Nivolumab (every 2 weeks at 240 mg) when given concurrently with the vaccine		
		·Colorectal carcinoma	And nivolumab (every 4 weeks at 480 mg) after vaccine treatment is completed		
**No. 3 enhancing immune cell content**					
αPD1-MSLN-CAR-T cells	Increase the number of targeted T cells	·Early I trial	3 + 3 dose escalation approach (1 × 10^5^ CAR-T cells/kg, 3 × 10^5^ CAR-T cells/kg, 1 × 10^6^ CAR-T cells/kg, and 3 × 10^6^ CAR-T cells/kg)	NCT04503980	It could promote immune cell infiltration in the cold CRC (pMMR/MSI-L, CMS2, and CMS3), which would produce a good effect combined with ICIs.
		·Solid tumors (colorectal cancer)			
CAR-T/TCR-T cells	Increase the number of targeted T cells	·Phase I and II trials	Receive CAR-T cell immunotherapy with several different specific chimeric antigen receptors	NCT03638206	
		·Malignancies (colorectal cancer)			

For different CRC subtypes, we divided the immunotherapy strategies into three categories: checkpoint inhibitors, enhancing tumor immunogenicity, and increasing the immune cell number in the TME. Ongoing clinical trials for different CRC strategies are shown in the table.

CRC, colorectal cancer; PD-1, programmed cell death 1; CTLA4, cytotoxic T lymphocyte antigen 4; dMMR, deficient DNA mismatch repair; MSI-H, high microsatellite instability; mCRC, metastatic colorectal cancer; VEGF, vascular endothelial growth factor; pMMR, proficient mismatch repair; MSS, microsatellite stability; DCs, dendritic cells; TAAs, tumor-associated antigens; CAR-T, chimeric antigen receptor-modified T; TME, tumor microenvironment.

## 3 Immune Checkpoint Inhibitor-Based Strategies for Colorectal Cancer Classified by the Mismatch Repair/Microsatellite Instability System

Genes involved in MMR regulate DNA MMR, and a loss of expression causes the accumulation of mismatches during DNA replication, resulting in MSI. Approximately 15% of CRC cases are caused by the MSI pathway (41). According to the different states of the MMR/MSI system, CRC is divided into two subsets. The DNA MMR system relies on some key genes, such as MLH1, MSH2, MSH6, PMS2, or MSH3, which correct mismatched, misinserted, or deleted bases in DNA (42, 43). If the gene repair function is inactivated due to MMR protein defects, these errors in DNA synthesis may freely and permanently integrate into the cellular DNA, resulting in MSI of CRC. As an important marker of MMR protein deficiency, MSI stability has become another major indicator to guide treatment strategies for patients with CRC (44–46). According to the degree of instability in detection markers, MSI is divided into MSI-H, MSI-L, and MSS. dMMR is lacking MMR proteins, which is mainly manifested as MSI-H. pMMR shows normal expression of MMR proteins and includes MSI-L and MSS.

In recent years, as in-depth studies on immune markers have been conducted, a large number of studies have shown that a high TMB predicts the efficacy of ICIs in the treatment of CRC (7, 39, 47). In the MMR/MSI classification system of CRC, patients with the dMMR/MSI-H subtype have a higher TMB, which might be associated with higher expression of neoantigens on MHC-I molecules (48). From The Cancer Genome Atlas (TCGA)-CRC cohort of 276 patients with CRC published in 2012, 44 (16%) CRC specimens showed a hypermutant phenotype (defined as greater than 12 TMB mutations per 106 bases). Meanwhile, 37 patients presented the MSI-H subtype of CRC (49). In addition, POLE mutant CRC usually presents with the pMMR-MSS phenotype, which also belongs to the hypermutant phenotype. The TMB in MSI-H and MSS is higher than that in MSI-L. All these subtypes of CRC are highly sensitive to ICI treatment strategies (50), indicating that the MMR/MSI classification system is also helpful in guiding decisions on immunotherapy strategies for patients with CRC. In this section, we distinguished patients with three subsets of CRC based on the MMR/MSI system and summarized the different ICI strategies for three of the CRC subtypes.

### 3.1 Immune Checkpoint Inhibitor-Based Strategies for the Deficient Mismatch Repair/High Microsatellite Instability Colorectal Cancer Subtype

As mentioned above, based on the MMR/MSI classification system, dMMR is mainly characterized by MSI-H, which is defined as instability at two or more sites that results in a large number of DNA replication errors, highlighted by genetic and accidental changes in the MMR gene (51). Through whole exome sequencing of the same number of CRC tumor cells, an average of 1,782 individual cell mutations were found in dMMR/MSI-H tumors, while only 73 individual cell mutations were detected in pMMR/MSS tumors (10). Since most of the dMMR/MSI-H CRC subtypes have a high TMB, ICIs and immunotherapy exert excellent therapeutic effects on patients with a high TMB (52). Therefore, ICI immunotherapy strategies have become the primary clinical treatment for patients with the dMMR/MSI-H subtype, including PD-1 inhibitor (pembrolizumab or nivolumab) monotherapy (19, 53), combination therapy with a PD-1 inhibitor (nivolumab), and CTLA4 inhibitor (ipilimumab) (54) and combination therapy with a PD-L1 inhibitor (atezolizumab) and anti-angiogenic vascular endothelial growth factor (VEGF) antibodies (bevacizumab) (55). For example, Overman enrolled 74 patients with the MSI-H subtype in a clinical study (NCT02060188) and treated them with nivolumab, a PD-1 inhibitor (56, 57). After treatment with nivolumab, approximately 68.9% of patients required longer than 12 weeks for disease control. In addition, eight patients (34.8%) experienced an immune response lasting longer than 12 months, indicating that nivolumab provides long-lasting response and disease control in patients with dMMR/MSI-H mCRC. More interestingly, in patients with the MSI-H subtype, the combination strategy incorporating ICIs appears to exert a greater antitumor effect. In 2018, Overman and colleagues further explored the therapeutic effect of the combination of the PD-1 inhibitor nivolumab and the CTLA4 inhibitor ipilimumab on MSI-H tumors (54). Among 119 patients, the combination inhibitor treatment achieved 80% effective tumor control at 12 weeks, and more than 94% of the immune response was sustained. All these data showed that the combination strategy of a PD-1 inhibitor and CTLA4 inhibitor results in a higher immune response rate and longer overall survival (OS) and progression-free survival (PFS) durations for patients with the MSI-H subtype, prompting the Food and Drug Administration (FDA) to approve nivolumab and ipilimumab as treatments for patients with the dMMR/MSI-H subtype (22). In conclusion, significant therapeutic efficacy has been documented for ICIs for the MSI-H subtype, providing a promising new treatment option for patients.

### 3.2 Immune Checkpoint Inhibitor-Based Strategies for the Proficient in Mismatch Repair/Microsatellite Instability Colorectal Cancer Subtype

Recently, a randomized trial was conducted with the PD-L1 and CTLA4 inhibitors durvalumab and tremelimumab, respectively, for supportive pMMR/MSS CRC therapy (58). One hundred eighty patients with CRC were divided into the D+T group (durvalumab (D) and tremelimumab (T)) and BSC group (best supportive care). Although the objective response rate (ORR) and PFS were similar between the two groups, the OS was improved in the D+T group, indicating that the survival time was extended using durvalumab and tremelimumab immunotherapy. This study is the first to combine a PD-L1 inhibitor and CTLA4 inhibitor to prolong the survival of patients with pMMR/MSS advanced refractory CRC, raising new hopes for immunotherapy for pMMR/MSS mCRC. However, the clinical benefit is limited to a small subset of patients with the pMMR/MSS CRC subtype, accounting for approximately 4% of all patients with CRC (59, 60). For pMMR/MSS CRC subtype, ICI treatment cannot achieve the best therapeutic effect. When MSI is present, tumor cells release many tumor-associated antigens (TAAs) that are normally located inside tumor membranes, which are then taken up and presented by antigen-presenting cells (APCs) located in the tumor immune microenvironment, enhancing the antitumor ability of T cells (61, 62). Nevertheless, for the pMMR/MSS subtype of CRC, the DNA structures are too stable to release TAAs, thus blocking immune system activation or inducing a failure of activated immune cells to recognize tumor cells. Therefore, some studies have indicated that PD-1 inhibitors are less effective in patients with pMMR/MSS CRC subtypes (10, 63–66). New therapeutic strategies are urgently needed to enhance tumor immunity in patients with pMMR/MSS CRC. To date, many studies have indicated that chemotherapy, molecular targeted therapy, and radiotherapy cause immunogenic cell death (ICD) in cancer cells (67). After ICD, tumor cells are exposed to a large number of TAAs, and damage-related molecular patterns and proinflammatory cytokines are released and effectively promote immune cell infiltration and activate APCs (such as dendritic cells (DCs) and macrophages). Then, DCs and macrophages gradually mature and cross-present tumor antigens after their uptake, resulting in antigen-specific immune responses to tumors (68). The immunogenic-based treatment strategy covers all associated antigens of tumor cells and minimizes the incidence of immune escape, thereby mediating a systemic immune response through site-specific *in situ* cell death (69–72). These theories might provide new immunotherapy strategies for patients with pMMR/MSS CRC presenting a low immune response.

In addition, a recent study identified a key mechanism of T-cell failure and resistance to checkpoint blockade in patients with MSS CRC. The depletion of tumor-infiltrating T cells and a simultaneous increase in VEGF-α levels were observed in MSS colorectal tumors, which might explain why human T cells deplete the associated transcriptional programming in a virulence-dependent manner (73–75). After VEGF-α was inhibited, T cells restored antitumor viability. The combination of a PD-1 inhibitor and VEGF-α inhibitor effectively restores the antitumor function of T cells in MSS CRC, achieving a better therapeutic effect (76).

### 3.3 Immune Checkpoint Inhibitor Strategies for the Proficient in Mismatch Repair/Low Microsatellite Instability Colorectal Cancer Subtype

For patients with the pMMR/MSI-L CRC subtype, the microsatellite stability of the cancer cells ranges between MSI-H and MSS subtypes. Therefore, this subtype has few specific tumor characteristics. To date, commonly used immunotherapy strategies for the MSI-L subtype are divided into three categories: CTLA4 inhibitors (ipilimumab), PD-1 inhibitors (pembrolizumab or nivolumab), and PD-L1 inhibitors (atezolizumab or durvalumab), as well as their combinations. Therefore, the combination of pembrolizumab and nivolumab and the combination of nivolumab and ipilimumab have been approved for the treatment of CRC by the FDA (77). ICIs are not absolutely effective against the MSI-L subtype. In a study published in 2015, Le et al. observed that pembrolizumab treatment did not exert favorable immune-mediated antitumor effects on patients with PMMR MSI-L tumors (10). In addition, in the study by Overman, 142 patients with pMMR/MSI-L had a limited response to immunotherapy. Only one of 20 patients exhibited an immune-mediated antitumor response to the combination of PD-1 and CTLA4 antibodies (54). In recent years, with the discovery of new ICI combination strategies, substantial progress has been achieved in improving the efficacy of combination immunotherapy for patients with this type of tumor. According to the study by Liu published in 2015, the combination of a PD-L1 inhibitor and MEK inhibitor showed significant efficacy in patients with pMMR/MSI-L CRC (78). The RAS–MAPK pathway is the intersection or the last common pathway of transmembrane transduction of growth signals by multiple membrane receptors. Activation is related to the reduction of tumor infiltration by T cells and directly promotes the proliferation of tumor cells. MEK is the downstream effector of the RAS–MAPK pathway. Mekinist, a MEK inhibitor, increases the tumor infiltration of CD4^+^ T lymphocytes but does not affect CD8^+^ T cells. More importantly, in murine models of KRAS-mutated CT26 colorectal tumors, when the two drugs were used together, more potent and long-lasting antitumor activity was observed than in mice treated with either of the two drugs alone (18, 78, 79).

Endocellular peptides are processed and expressed on major histocompatibility complex class I (MHC I) molecules on the surface of almost all human cells, including cancer cells. This type of peptide is specifically recognized by T-cell receptors (TCRs) (18). The response of T cells is modulated by a series of coinhibitory or costimulatory signals. Among them, the membrane-binding ligands CD80 and CD86 of the B7 family bind to the costimulatory protein CD28, especially in activated T cells, resulting in an interaction with CTLA4 and the inhibition of T-cell activity (80). Similarly, membrane-bound PD-L1 and programmed cell death 2 ligand (PD-L2) bind to PD-1 to further incapacitate T cells and induce apoptosis (81–83). When these inhibitor signals are antagonized by the corresponding antibodies, T cells are activated and produce an antitumor effect. However, the T-cell content appears to be deficient in the MSI-L subtype, which may be the root cause of the poor response of this type to immunotherapy (56). In addition, the combination of a PD-1 inhibitor and other modulators of immune checkpoints, such as CTLA4, may benefit a small subset of patients with pMMR/MSI-L tumors. This benefit might be associated with the low microsatellite instability of the tumor cells themselves. Due to its poor specificity, this subtype displays low sensitivity to ICIs. Currently, few studies have been conducted on immunotherapy for this type of CRC, and more effective strategies should be designed that mainly focus on approach to increase the infiltration of immune cells into pMMR/MSI-L tumors.

## 4 Immune Checkpoint Inhibitor-Based Strategies for Hot/Cold Colorectal Cancer Classified Based on the Degree of Immune Cell Infiltration

As a highly heterogeneous malignant tumor disease, the biological behaviors of any two different CRC lesions vary widely in terms of the genetics and epigenetics of different lesions (84). Therefore, a method that can classify CRC according to tumor heterogeneity at the molecular biological level in the current era of precision treatment development is urgently needed. Consensus molecular subgroups (CMS) are a consensus classification based on cancer gene expression proposed by Guinney (85). Due to the extensive chromosomal alterations and dMMRs in CRC, genetic heterogeneity exists among different CRC cells. Based on pathology and molecular biological data from 3,000 patients with CRC, Guinney classified patients with CRC into four subtypes: CMS1, microsatellite unstable immunotype (14%), which is characterized by high MSI-H mutations and exhibits both BRAF mutations and strong immune cell infiltration; CMS2, the most common type (37%), which is characterized by activation of the WNT and MYC pathways and chromosomal instability; CMS3, the metabolic type (13%), which is mainly characterized by mutations in KRAS, mixed MSI status, and abnormal metabolic pathways; and CMS4, a mesenchymal subtype (3%), is characterized by activation of transforming factor TGF-β and enhanced angiogenesis, interstitial infiltration, and inflammatory infiltration (20, 85, 86). Among the four subtypes, the CMS1 and CMS4 subtypes are characterized by more extensive lymphocyte infiltration and a higher distribution of inflammatory cytokines around the tumor, while the CMS2 and CMS3 subtypes exhibit almost no lymphocyte or inflammatory cell infiltration. However, in the TME, the number of infiltrated immune cells directly modulates the effect of ICIs. As a result, the four subtypes of CRC based on the CMS system were artificially divided into two types, hot CRC and cold CRC, according to the presence of lymphocyte infiltration and the inflammatory environment around the tumor (39).

### 4.1 Characteristics of Hot Colorectal Cancer and Immune Checkpoint Inhibitor Therapy Strategies

The TME of hot CRC contains many lymphocytes and inflammatory infiltrates, which mainly include the CMS1 and CMS4 subtypes. As the main antitumor cells, T cells are abundant in hot tumors and are more easily activated, and patients with these subtypes are more likely to benefit from immunotherapy (86). Although a large number of immune cells are present around both the CMS1 and CMS4 subtypes, the CMS4 mesenchymal subtype seems to be more prone to an adverse inflammatory immunophenotype characterized by the activation of transforming factor TGF-β and enhanced tumor angiogenesis, tumor growth, and metastasis. The malignant inflammatory environment will block the antitumor effect of immune cells, resulting in immunosuppression. Therefore, different immunotherapy strategies for the two subtypes of hot tumors are expected to obtain the maximum therapeutic benefits from personalized immunotherapy.

#### 4.1.1 CMS1 Microsatellite Unstable Immunotype

CMS1, also known as the MSI-like subtype, is the main potential beneficiary of immunotherapy for CRC. In the TME of the CMS1 subtype, the infiltration of a large number of immune cells, such as T cells, and a high BRAF gene mutation burden increase the effectiveness of immunotherapy. In 2016, Becht et al. showed that when a large number of invasive T cells, especially cytotoxic CD8^+^ T cells, accumulate in the TME, patients experience longer PFS and OS (21, 87). The authors suggested that the effective release of TAAs by CRC tumor cells induces a locally adaptive immune response (88). Meanwhile, they also detected large numbers of infiltrating T and B cells in tumors of the CMS1 subtype, which produced a strong guarantee for treatment strategies of ICIs such as PD-1/PD-L1 inhibitors. Tumor cells of the CMS1 subtype overexpress CXCL9, CXCL10 (specific chemokines that recruit T cells), and CXCL13 (protein that recruits B cells) and release IFNG and IL-15 (89). These characteristics promote the recruitment and activation of immune cells such as APCs or T cells, which are closely associated with a good prognosis in patients with CMS1 (87, 89, 90). In addition, some studies found that PD-1 is also expressed at high levels on the surface of CMS1 tumors. Therefore, for CMS1 tumors with a high level of T-cell infiltration, the use of ICIs such as PD-1 inhibitors might effectively assist infiltrating T cells in escaping the immune suppression of tumor cells, thus activating an effective antitumor immune response (13, 91). In general, the CMS1 subtype of CRC with a high level of immune cell infiltration has a better immune response and immunotherapy with ICI exerting a better antitumor effect (92).

#### 4.1.2 CMS4 Mesenchymal Subtype

CMS4 is the second largest subtype, accounting for approximately 23% of the total number of CRC cases (85). The main manifestations are the activation of transforming factor TGF-β, enhanced angiogenesis, interstitial infiltration, and inflammatory infiltration. However, unlike CMS1, CMS4 has an adverse inflammatory immunophenotype, which leads to a poor immune microenvironment, and active T cells are unable to kill tumor cells. The antitumor response of potential immune cells in the TME of CMS4 is potentially blocked by adverse inflammatory infiltration in the stroma, thus inhibiting the immune response to cancer cells. Therefore, little therapeutic strategies by using ICIs were studied in CMS4 subtype. According to the underlying mechanism of immunosuppression, more newly approaches were explored to reactivate antitumor immunity with the aim of determining the main mechanism of immunosuppression in patients with CMS4, described as follows: 1) by changing the immunosuppressive environment of the TME, immune cells are remodeled, and the antitumor functions of macrophages, DCs, and T cells are reactivated to transform the TME of CMS4 into a hot tumor-like subtype (93). 2) Significant infiltration of fibroblasts and innate immune cells is observed in CMS4, and the increase in TGF-β signaling exerts a significant inhibitory effect on immune cells. Therefore, the use of selective TGF-β inhibitors in combination with ICIs might be useful for immunotherapy in patients with CMS4 (94, 95). 3) CMS4 promotes angiogenesis and increased expression of VEGF-related factors, such as FGF, in the microenvironment, which might promote tumor growth. Among them, the proangiogenic molecule VEGF has been shown to play an important role in promoting tumor development in the immunosuppressive microenvironment. Methods to eliminate VEGF or reduce its expression and release have become another research direction. Currently, many clinical studies are investigating the efficacy of VEGF inhibitors for CRC. For example, many clinical trials have attempted to use bevacizumab (VEGF inhibitor) in combination with classic chemotherapy regimens, such as FOLFOX and FOLFIRI (NCT03635021 and NCT02339116), which exert a better antitumor effect. In a phase III clinical trial published in 2020, the authors reported a significantly longer PFS in the combination treatment group than in the control group (96). The results indicated that initial treatment with FOLFOXIRI plus bevacizumab followed by the reintroduction of the same regimen after disease progression seems to be a preferable therapeutic strategy and has an acceptable safety profile. However, the real function of the combination strategy also must be confirmed in appropriate, larger randomized clinical trials.

### 4.2 Characteristics of Cold Colorectal Cancer and Immune Checkpoint Inhibitor Therapy Strategies

CMS2 and CMS3 subtypes are the main representative types of cold CRC tumors. The CMS2 subtype is the most common subtype, accounting for approximately 37% of all CRC cases (85). The subtype is mainly gene-specific, showing activation of WNT and MYC pathways and chromosomal instability. CMS3 mainly presents as an epithelial neoplasm. This subtype is characterized by KRAS gene mutations, a mixed MSI status and abnormal metabolic pathways. Both of these types exhibit gene-specific expression and methylation abnormalities. It has been explored that ICIs drugs alone have poor efficacy, which is related to the immunosuppressive nature of cold CRC. Currently, a variety of immunotherapeutic strategies have been developed, such as oncolytic virus, cytokine therapy, CAR-T therapy, and passive immunotherapy against TAAs (97), which have contributed to the immune-activated antitumor therapy of cold tumors.

The main characteristic of the CMS2 and CMS3 subtypes is the lack of tumor immunogenicity in the TME (98). The immunosuppression mechanism is different from that of hot tumors. Therefore, methods to activate the tumor cells of CMS2 and CMS3 to release TAAs are the starting point of immunotherapy for cold tumors. ICD is mediated by the release of TAAs when tumor cells undergo apoptosis, which stimulates long-lasting antitumor immune effects in the body (99). ICD of tumor cells substantially improves the low immunogenic microenvironment of cold tumors and promotes immune activation. Chemotherapy is one of the main methods to induce ICD in tumor cells. Some anthracyclines and oxaliplatin not only induce tumor cell apoptosis but also induce ICD (67). When TAAs are released, APCs such as DCs and macrophages are activated, along with the activation of T cells (100, 101). In addition, the efficacy of chemotherapy combined with ICIs becomes an important therapeutic strategy for cold CRC and has been well established. In 2017, chemotherapy combined with pembrolizumab (Keytruda) was approved by the FDA as a first-line treatment for metastatic non-small cell lung cancer (NSCLC) (102, 103). The effective rate of Keytruda combined with chemotherapy was 55%, while the effective rate of chemotherapy alone was only 29%. Combination therapy reduced the risk of disease progression. Since then, the FDA approved Keytruda in combination with pemetrexed and platinum-based chemotherapy as a first-line treatment for metastatic non-squamous NSCLC in patients without EGFR and ALK cancer genetic variants in August 2018 and approved Keytruda in combination with standard chemotherapy (carboplatin and paclitaxel/albumin paclitaxel) as a first-line treatment for squamous NSCLC in October 2018.

In addition, lower immune cell infiltration is another major factor contributing to a poor immune response (104), which is also a reason for ineffective ICIs. The low immune cell infiltration of cold tumors results in the insufficient effect of the activated immune system on eradicating the tumor tissue. Therefore, approaches that promote the enrichment of immune cells and activate the strong and lasting immune antitumor response have become the main challenge to overcome in immunotherapy for cold CRC. In a study presented at the ASCO-SITC Symposium on Clinical Immuno-Oncology in 2020, Shen and colleagues found that the methylation and expression levels of ARHGAP9, TBX21 (T-bet), and LAG3 genes were associated with the infiltration of CTLs (105). In CMS2 tumor cells with low T-cell infiltration, the expression of ARHGAP9 and T-bet genes was downregulated, and the methylation level was low. The expression of ARHGAP9, T-bet, FML1, HLA-DPB1, and STX11 genes in the CMS3 subtype was also downregulated, and the methylation level was low (105). However, in the CMS1 subtype with high infiltration of immune cells, the expression level and methylation level of T-bet gene were higher. Therefore, the expression levels of these genes differ between CMS subtypes, and the methylation level is related to the extent of CTL infiltration. T-bet gene was the only gene that was strongly correlated with CTL infiltration, which might directly reflect the degree of T-cell infiltration in patients with CRC. Therefore, the T-bet protein is likely to be a key regulator of T-cell infiltration in CRC (106). An increase in the methylation level of T-bet gene in the CMS2 and CMS3 subtypes and upregulation of the expression level of T-bet gene have become effective strategies for cold tumor immunotherapy.

## 5 Other Types of Immunotherapies Instead of Immune Checkpoint Inhibitors as Colorectal Cancer Treatment Strategies

### 5.1 Chemoimmunotherapy Strategy

ICIs has always been the most mature and effective immunotherapy for CRC. However, it appears to be limited for CRC subtypes with low immunogenicity or low immune cell infiltration. Therefore, immunotherapy by using ICIs alone is not effective in some specific CRC subtypes. Chemoimmunotherapy has become an important therapeutic strategy in the treatment of CRC. CAVE-Colon study has been proposed in recent years (NCT04561336). The researcher found that the combination strategy of cetuximab, an EGFR-targeted drug, and avelumab, an ICI drug, would significantly improve survival of CRC patients. Cetuximab improved the T-cell activity by increasing immune cell invasion and inducing APC maturation, while ICIs eliminated the tumor immunosuppressive effect on T cells.

In addition, in order to further improve the survival of CRC patients treated by FOLFOX or FOLFIRI strategies, a new GOLFIG was developed (107, 108). This kind of strategy combined low-dose recombinant interleukin-2 (rIL-2) and granulocyte macrophage colony-stimulating factor (GM-CSF) based on FOLFOX and gemcitabine, a chemotherapy drug. In the GOLFLG strategy, multiple chemotherapy drugs can successfully destroy tumor cells in large quantities, while rIL-2 and GM-CSF would promote the activation of APCs, then activate CTLs, and then accurately destroy the remaining tumor cells (109, 110). Correale et al. firstly explored the effect of FOLFIG trials. They found the remission rate (RR) and disease control rate (DCR) of patients with mCRC were higher than those of FOLFOX strategy, indicating significant immune response and antitumor activities (111). In addition, PFS was significantly lower in patients treated with GOLFIG than with FOLFOX, indicating GOLFIG could effectively reduce the recurrence rate (112, 113). Chemoimmunotherapy has broadened the scope of application of ICIs and maximized the antitumor effect.

### 5.2 Oncolytic Virus and Chimeric Antigen Receptor-Modified T-Cell Immunotherapy for Colorectal Cancer

**Figure 2:**
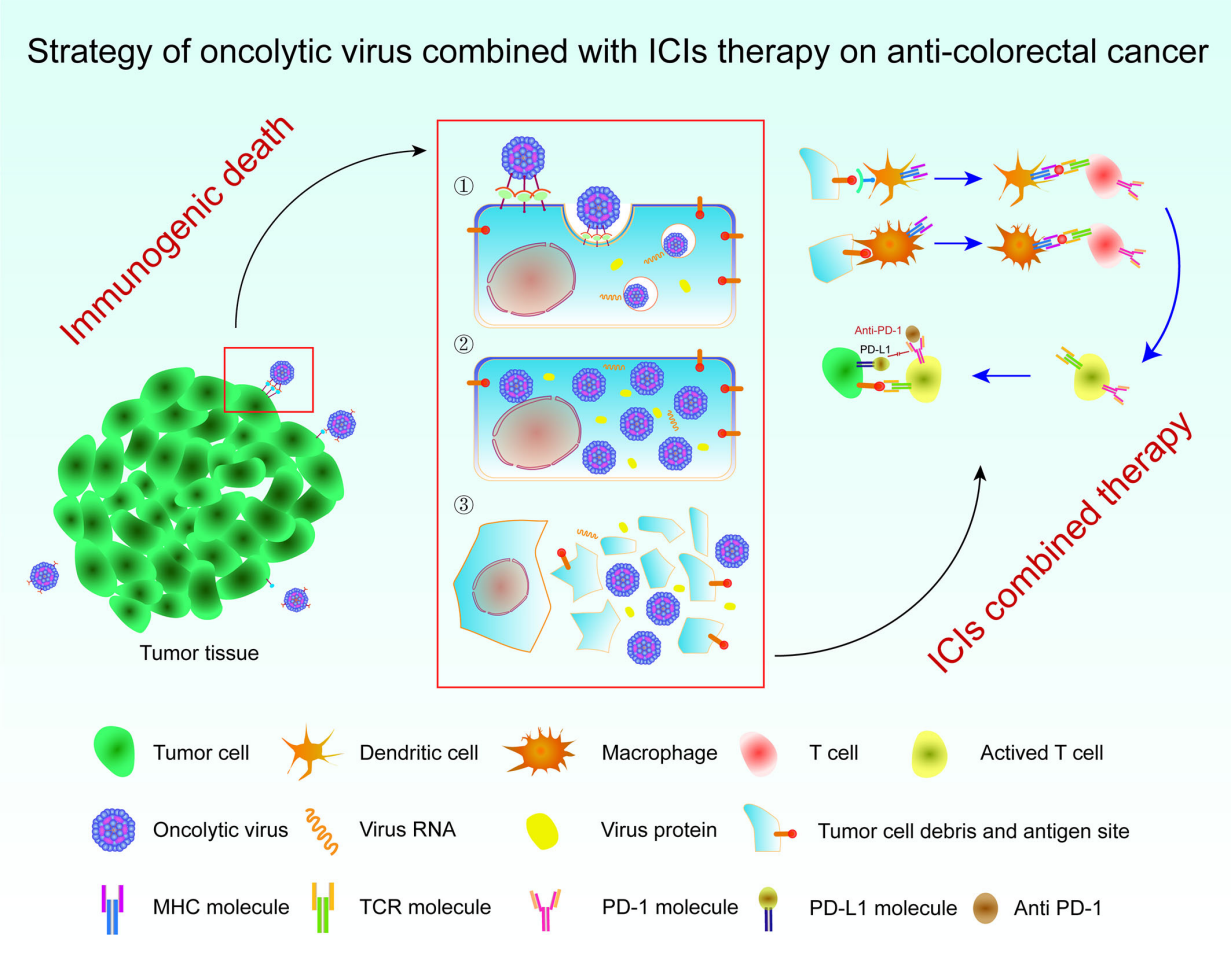
Strategy of an oncolytic virus combined with ICI therapy for colorectal cancer. Immunogenic cell death occurs in tumor cells upon the action of oncolytic viruses, which release TAAs to activate APCs and then promote T cells to attack tumor cells. The combination of an oncolytic virus with ICIs removes the inhibition of T cells by tumor cells through the immune checkpoint. The tumor tissue is then removed to the maximum extent possible. ICI, immune checkpoint inhibitor; TAAs, tumor-associated antigens; APCs, antigen-presenting cells.

Oncolytic virus immunotherapy is a promising approach for the treatment of solid tumors and has the ability to selectively replicate the tumor, achieve ideal immunogenicity, and deliver foreign genes to the tumor in a targeted manner (114, 115). In 1991, Martuza et al. showed that transgenic HSV was an effective treatment for glioblastoma (116). Since then, oncolytic virus therapy based on HSV has been developed. The antitumor strategy of oncolytic viruses is mainly mediated by the genetic modification of some of the less virulent viruses existing in nature. These approaches use the inactivation or defect of tumor suppressor genes in target cells to produce therapeutic oncolytic viruses with a specific recognition ability, which selectively infect tumor cells, then replicate in large numbers, and eventually destroy tumor cells. Meanwhile, damaged tumor cells release a large number of TAAs, and thus the immunogenic death of tumor cells is induced to attract more immune cells and clear the tumor tissue (116). In recent decades, oncolytic virus therapy has attracted increasing attention and achieved great progress in related research. The oncolytic vaccinia virus (VV) treatment strategy has been shown to transform cold tumors of the CRC type into hot tumors by promoting the infiltration of immune cells, resulting in effective antitumor immunity (117, 118). However, the therapeutic potential of oncolytic viruses is also affected by the immunosuppression of immune cells by tumors, such as immune checkpoints. According to the study published by Wei, compared with oncolytic VV therapy alone, the strategy of VV combined with monoclonal antibody against T-cell immunoglobulin and ITIM domain (TIGIT) exerts better effects on reducing the tumor burden and prolonging survival (119). They found that the number of CD8^+^ T cells increased and the microvascular density decreased in the TME of CRC using the VV-ATIGIT strategy for immunohistochemistry, which proved that the combination strategy significantly inhibited tumor growth in mice with colon cancer. In conclusion, oncolytic viruses combined with ICIs may be a new direction for the treatment of CRC (**Figure 2**) (119).

For CMS2 and CMS3 CRC subtypes, the low level of immune cell infiltration is the main factor contributing to a poor immune response. The emergence of CAR-T cell therapy has become the main and precise treatment strategy to solve this difficulty. The CAR-T strategy is a classic adoptive cellular immunotherapy that kills tumor cells by injecting genetically modified and amplified immune effector T cells (120). CAR-T immunotherapy has many advantages, such as strong specificity, strong targeting, a high fatality rate, few side effects, and no drug resistance (119). For some CRC subtypes with low immune cell infiltration, CAR-T therapy with high efficiency and specific targeting of tumors is undoubtedly an appropriate immunotherapy strategy. Carcinoembryonic antigen (CEA) is a sensitive biomarker for gastrointestinal tumors that is widely expressed in CRC tissues and serum and not expressed in many other normal tissues or organisms (121), making it an effective target for CAR-T therapy. In 2017, Zhang et al. conducted a phase I clinical trial of systemic intravenous CAR-T therapy for CRC with high positive CEA expression (NCT02349724). In the trial, five stepped-up CAR-T cell doses were administered to 10 patients with CRC, and no serious adverse events associated with CAR-T therapy were observed during follow-up. Seven of the 10 patients had progressed at the time of previous treatment but were stable after CAR-T treatment. Imaging analysis showed that tumors shrank after treatment in two patients. After long-term observation, the serum CEA level decreased significantly in most patients. In addition, CAR-T persisted in the peripheral blood of patients who received high doses of CAR-T. More importantly, proliferation of CAR-T was observed in the trial, especially in patients following the second CAR-T treatment. CEA-targeted CAR-T therapy was well tolerated by patients with CEA-positive CRC, even at high doses. Some efficacy was observed in the majority of patients who received the therapy (17).

In 2019, Ying et al. loaded the CD19 truncated (CD19T) gene in an oncolytic virus to increase the expression of the CD19 antigen on the tumor cell membrane surface by taking advantage of the transgenic transmission potential of the oncolytic virus (122). Meanwhile, they combined CAR-T immunotherapy with the chimeric CD19 gene to enhance the specific targeting effect of CAR-T. The combination of the oncolytic virus and truncated CD19 (OV-CD19T) specifically replicated and expressed CD19T antigen in different solid tumor cells. The combination of OV-CD19T and CD19 CAR-T therapy is a safe and effective treatment for multiple solid tumors both *in vitro* and *in vivo*, without causing neurotoxicity or severe cytokine release syndrome (CRS), suggesting long-lasting tumor killing effects (122). In the next year, Priceman further explored a novel and effective combination immunotherapy based on the combination method (123). They used a genetically engineered oncolytic virus to overexpress the CD19 antigen on the surface of tumor cell membranes, thereby enhancing the antitumor response of CD19 CAR-T therapy to solid tumors. CD19 has long been an ideal target for CAR-T against hematological malignancies due to its highly restricted expression on B cells (124–126). CD19-mediated CAR-T therapy has been approved by the FDA for B-cell malignancies. However, in solid tumors, the coexpression of most tumor antigens in normal tissues limits the potential of CAR-T in solid tumors. The strategy of introducing specific targets into solid tumors to promote CAR-T immune activation might advance the suitability of CAR-T therapy for solid tumors.

### 5.3 Cancer Immunization Vaccine

The biological process of ideal immune-activated antitumor therapy is that ICD tumor cells release TAAs, damage-associated molecular patterns, and proinflammatory cytokines, which function as an alarm to effectively promote immune cell infiltration and activation. APCs promote cross-presentation of tumor antigens, triggering a wide range of tumor antigen-specific immune responses (127). T cells, the terminal killer cells of immune antitumor cells, are of decisive significance for immune efficacy. However, cold CRC tumors have low immunogenicity or less T-cell infiltration in the TME, which may be due to the presence of initiation defects or the absence of high-affinity T cells. Therapeutic cancer vaccines with TAAs or tumor-specific antigens (TSAs) might directly stimulate the immune system and induce an immune response by delivering these antigens directly to professional APCs, thus activating CD4^+^ and CD8^+^ T cells to kill tumors (128, 129). Many cancer vaccines have been developed, including cell vaccines, nucleic acid vaccines, protein peptide vaccines, and genetically engineered vaccines (130–134). Adoptive cell immunotherapy (ATT) is a major cellular vaccine strategy and a novel approach to TSA presentation. When the immune cell antigen load is low or the relevant tumor antigen load is not available, ATT might achieve better tumor targeting and killing effects by directly overexpressing tumor cell antigen antibodies in immune cells (135). For example, CD8^+^ T cells targeting k-ras or TP53 mutations have been identified (136, 137). In addition, recent studies have shown that circulating PD-1-positive lymphocytes recognize neoantigens in human digestive tract tumors (32, 138).

Thymidylate synthase poly-epitope-peptide (TSPP) is an anticancer poly-epitope peptide vaccine, which can promote the promote cross-presentation of tumor antigens through APCs and trigger a highly specific immune response with multi-antigen specificity (139). TSPP has shown good antitumor activity in preclinical studies. However, the antitumor effect of TSPP is significantly influenced by the TME such as inflammation and immune response. K-ras and IL17/A have been found to be important factors to predict the resistance of TSPP. Patients with k-ras mutation have no statistically significant inflammatory markers and also have poorer immune response (139). IL17/A, as an inflammatory cytokine independent of k-ras status, could amplify and enhance the cytotoxic effects of CTLs at CRC tumor sites (140). By predicting k-ras mutation and IL-17/A level, the immunoreactivity and antitumor efficacy of immunotherapy like TSPP vaccine against CRC could be evaluated.

In addition, the immunotherapy response is usually associated with the TMB, and patients with dMMR/MSI-H have higher genetic mutation characteristics (52). Therefore, vaccination against the associated antigens of specific genetic mutations has proven effective against this type. In 2017, Maletzki et al. constructed a mouse model of dMMR induced by knockout of MLH1. After the treatment dMMR cancer vaccine was administered to the mice, the OS time was prolonged, and the tumor mutation load was reduced, indicating that the specific vaccine is a viable option for the treatment of the dMMR subtype (141, 142). Similarly, human clinical trials of therapeutic cancer vaccines have shown good results based on the MSI status (143). The main question to be answered in order to promote increased immune efficacy is whether the combined ICI immunotherapy and vaccine strategy is more effective than ICIs alone in dMMR/MSI-H CRC or whether it is capable of causing a response in pMMR/MSI-L CRC that does not respond to ICIs.

### 5.4 Immune Adjuvant

The TME is complex and changeable, and the immune response is affected by many factors. Different types of CRC are heterogeneous. Even for the same solid tumor, a large difference in gene or protein expression may be observed. Therefore, extensive clearance of tumor cells is difficult to achieve with a single immunotherapy (144). Immune adjuvant, a non-specific immunoproliferative agent, is an adjuvant that effectively activates the immune response to antigens or changes the type of immune response, which is important for enhancing the efficacy of immunotherapy for CRC. At present, immune adjuvants are mainly divided into biological and abiotic adjuvants, and the latter exerts a better effect on immune sensitization. In a phase I clinical trial, Hou used Mn^2+^ combined with an anti-PD-1 antibody to safely enhance the antitumor efficacy of treatment for advanced mCRC (145). A lack of Mn^2+^ promotes tumor growth. When B16F10 melanoma cells were implanted in Mn^2+^-deficient mice, at least 2.5 * 10^3^ cells were needed. However, seven times more tumor cells were needed in normal mice to achieve 100% tumor incidence. Moreover, the tumor volume and weight of Mn^2+^-deficient mice were significantly increased. Tumor infiltration of CD4^+^ and CD8^+^ T cells was significantly decreased (146, 147). After the Mn^2+^ injection, tumor growth was inhibited. At the same time, Mn^2+^ injection on one side of the tumor also inhibited tumor growth on the other side, suggesting a systemic antitumor response. Moreover, Mn^2+^ promoted DC maturation and enhanced antitumor effects through the cGAS–STING pathway, which also exerts an antitumor effect on patients with immune-tolerant tumors. From 2018 to 2019, Lv et al. initiated a phase I clinical trial on the effect of Mn^2+^ on advanced mCRC. In this study, a MnCl_2_ solution was administered intranasally or inhaled. The authors documented a clinical RR of 45.5% and a tumor control rate of 90.9% after treatment with the MnCl_2_ solution (148). More importantly, five patients who had previously failed to respond to anti-PD-1 antibodies or chemotherapy in combination with radiation showed good DCRs during the treatment, suggesting that Mn^2+^ restored immunotherapeutic efficacy in immune treatment-resistant patients. As an inorganic adjuvant, Mn^2+^ has undoubtedly become the most important immune sensitizer for immunotherapy of CRC. It potentially activates the immune response of patients with immune treatment-resistant CRC, providing a new and more effective therapeutic strategy (149).

## 6 Development Prospects and Challenges of Immune Checkpoint Inhibitors for Colorectal Cancer

As an effective treatment strategy following surgery, radiotherapy, chemotherapy, and targeted therapy, ICI immunotherapy strategies displays powerful antitumor efficacy and strong therapeutic potential in the treatment of CRC tumors. However, as an emerging treatment, many challenges remain in terms of safety and efficacy. For ICI treatment strategies, although good therapeutic effect has been shown in part of CRC subtypes, it is not ideal for some other CRC subtypes such as pMMR/MSS and dMMR/MSI-L. More new ICI combination strategies need to be developed by exploring their specific mechanisms. On the other hand, due to the heterogeneity of solid tumors and the external microenvironment, the efficacy of immunotherapy for solid tumors is not as expected (150). Low clinical target response rates and the risk of autoimmune diseases remain major limitations. An overactive immune system during treatment might cause severe adverse reactions in patients with CRC (151). In recent years, more combination therapies for ICIs have been developed, which solved some limitations of ICIs only for CRC therapy. For example, ICIs combined with chemotherapy could improve the immunogenicity of CRC subtypes. ICIs combined with CAR-T cell therapy could improve T-cell enrichment at the tumor site, exerting synergistic effects. However, although more CRC immunotherapy strategies are being developed, there is still no detailed classification targeted at the characteristics of CRC immune environment. A more meaningful approach would be to formulate the classification based on the different immune response characteristics of CRC. First, for the subtypes with high immune cell infiltration (dMMR/MSI-H, CMS1, and CMS4), treatment strategies for CRC could be modified according to the main molecular mechanisms of immunosuppression between tumor cells and immune cells, such as immune checkpoints. For example, patients with dMMR/MSI-H CRC have benefited the most from ICI immunotherapy. In addition, for patients with CMS4, TGF-β is a major signal that enhances tumor metastasis (152). Preclinical models suggest that TGF-β suppression resets immune rejection signals and may restore susceptibility to checkpoint suppression, suggesting that it represents a good target for future CRC immunotherapy research. Second, for low immune cell-infiltrating tumor subtypes (pMMR/MSS, dMMR/MSI-L, CMS2, and CMS3), improving the immunogenicity of the tumor while increasing the targeting of the immune system and improving immune cell invasion have become the most important immunotherapy strategies instead of using ICIs. Promising treatment options for patients with pMMR/MSS CRC include chemotherapy, targeted therapy, oncolytic virus, local ablation, and the combination of TLR agonists with checkpoint inhibitors. Although treatment strategies have shown good efficacy in early clinical or preclinical studies, they have not yet been used in clinical trials. In conclusion, as the most promising antitumor therapy currently available, immunotherapy has great potential to be explored as a therapeutic strategy for CRC, which may bring new hope to patients with CRC in the future.

## Author Contributions

RH conceived of the presented idea and researched the background of the study. RH prepared the figures and tables. RH, MJ, YL, WY, and XZ wrote the manuscript. RH, MJ, and CZ checked the manuscript. All authors contributed to the article and approved the submitted version.

## Funding

The authors thank the financial support by the transverse project “Studies on the effect and mechanism of GSG2 in the occurrence and development of colon cancer” (No. Z0S0001).

## Conflict of Interest

The authors declare that the research was conducted in the absence of any commercial or financial relationships that could be construed as a potential conflict of interest.

## Publisher’s Note

All claims expressed in this article are solely those of the authors and do not necessarily represent those of their affiliated organizations, or those of the publisher, the editors and the reviewers. Any product that may be evaluated in this article, or claim that may be made by its manufacturer, is not guaranteed or endorsed by the publisher.
